# Supplementary material for: Novel Salmonella Phage, vB_Sen_STGO-35-1, Characterization and Evaluation in Chicken Meat
Source: Microorganisms. 2022 Mar 12;10(3):606. doi: 10.3390/microorganisms10030606 (PMC8954984; doi:10.3390/microorganisms10030606)
Supplement: Supplementary file 1 [file microorganisms-10-00606-s001.zip › Figure S/Figure S1.pdf]

A. Alignment of amino acid sequences present on Tailspike (gp65) STGO 35-1 phage and Salmonella phage P22 tail-spike 2FNA/A, was analysis by using CLUSTAL O version 1.2.4 (Sievers and Higgins, 2014).

```
pdb|2VFN|A      ------------------------------------------------------------ 0
Tailspike       MATTPTSLPIPSEDPRDLKFNAGKFDEVMTSDAHYYVDRFGVKRWTIAGFQYTAEEAIRA 60

pdb|2VFN|A      ------------------------------------------------------------ 0
Tailspike       YGYITMDSFEDGATLTLPNQVLRYEATGEYYRWDGAFPKAVAAGSTPASTGGVGLGAWIS 120

pdb|2VFN|A      MDPDQYSIEADKKFKYSAKLSDYPTLQDAASAAVDGLLIDRDYNFYGGETVDFGGKVLTI 60
Tailspike       VGDAAFRQEANKKFKYSVKLSDYSTLQEAATAAVDGVLIDRDYTFTDNETVDFGGKVLTI 180
                :.   :  **:******.***** ***:**:*****:******.* ..************

pdb|2VFN|A      ECKAKFIGDGNLIFTKLGKGSRIAGVFMESTTTPWVIKPWTDDNQWLTDAAAVVATLKQS 120
Tailspike       DCKAKFIGDGNLTFTNLGAGSLVNSPYMESATTPWVIKPWTDDNQWITDPAAVVATLKQS 240
                :*********** **:** ** : . :***:***************:** **********

pdb|2VFN|A      KTDGYQPTVSDYVKFPGIETLLPPNAKGQNITSTLEIRECIGVEVHRASGLMAGFLFRGC 180
Tailspike       KTDGYQPTVNDYAKFPGIESLLPPEAKDQNISSVLEIRECTGVEVHRASGLMACFLFRRC 300
                *********.**.******:****:**.***:*.****** ************ **** *

pdb|2VFN|A      HFCKMVDANNPSGGKDGIITFENLSGDWGKGNYVIGGRTSYGSVSSAQFLRNNGGFERDG 240
Tailspike       HFCKMIDADNPIGGKDGVITFENLSGDWGKGNYVIGGRTSYGSVSSAQFLRNNGGFARDG 360
                *****:**:** *****:************************************** ***

pdb|2VFN|A      GVIGFTSYRAGESGVKTWQGTVGSTTSRNYNLQFRDSVVIYPVWDGFDLGADTDMNPELD 300
Tailspike       GVIGFTSYRAGESGVKTWQGSVGSSTARNYNLQFRDSVVLYPVWEGFDLGADTDMNPEDD 420
                ********************:***:*:************:****:************* *

pdb|2VFN|A      RPGDYPITQYPLHQLPLNHLIDNLLVRGALGVGFGMDGKGMYVSNITVEDCAGSGAYLLT 360
Tailspike       RPGDFPFSQYPVHMLPLNHLIDNLLVRGSLGVGFGMDGKGLYVSNITVEDCAGSGAYILA 480
                ****:*::***:* **************:***********:****************:*:

pdb|2VFN|A      HESVFTNIAIIDTNTKDFQANQIYISGACRVNGLRLIGIRSTDGQSLTIDAPNSTVSGIT 420
Tailspike       HETVFTNIAIIDTNTKNFPANQIYISGACRVNGLRLVGIRSTNEQGLTIDAPNSTVSGIT 540
                **:*************:* *****************:*****: *.**************

pdb|2VFN|A      GMVDPSRINVANLAEEGLGNIRANSFGYDSAAIKLRIHKLSKTLDSGALYSHINGGAGSG 480
Tailspike       GLVDPSRINVANLADEGLGNTRINSFNNDSAALQLRIHKLTKTLDSGAVYSHINGGPGSG 600
                *:************:***** * ***. ****::******:*******:******* ***

pdb|2VFN|A      SAYTQLTAISGSTPDAVSLKVNHKDCRGAEIPFVPDIASDDFIKDSSCFLPYWENNSTSL 540
Tailspike       SAWTEITAISGSLPDAVSLKINRGDYRAVEIPVAMSPLPENAVRDIGSISMYLEG--DSL 658
                **:*::****** *******:*: * *..***.. .   :: ::* ..:  * *.   **

pdb|2VFN|A      KALVKKPNGELVRLTLATLL      560
Tailspike       KALVRRADGSYTRLTLA---      675
                ****:: :*. .*****
```

Match sequence (*) marked in red and in blue different sequence.

B. Conserved domains on PhageP22-tail [lcl|Query_16388] (Marchler-Bauer et al., 2015).

Name	Accession	Description	Interval	E-value
PhageP22-tail	pfam09251	Salmonella phage P22 tail-spike	79-274	0e+00

Pssm-ID: 312676 **Cd Length:** 550 **Bit Score:** 632.91 **E-value:** 0e+00

```
                           10        20        30        40        50        60        70        80
                   ....*....|....*....|....*....|....*....|....*....|....*....|....*....|....*....|
Query_16388      6 FKYSVKLSDYSTLQEAATAAVDGVLIDRDYTFTDNETVDFGGKVLTIDCKAKFIGDGNLTFTNLGAGSLVNSPYMESATT 85
Cdd:pfam09251    7 FKYSVKLSDYPTLQDAASAAVDGLLIDRDYNFYGGETVDFGGKVLTIECKAKFIGDGNLIFTKLGKGSRIAGVFMESTTT 86
                           90       100       110       120       130       140       150       160
                   ....*....|....*....|....*....|....*....|....*....|....*....|....*....|....*....|
Query_16388     86 PWVIKPWTDDNQWITDPAAVVATLKQSKTDGYQPTVNDYAKFPGIESLLPPEAKDQNISSVLEIRECTGVEVHRASGLMA 165
Cdd:pfam09251   87 PWVIKPWTDDNQWLTDAAAVVATLKQSKTDGYQPTVSDYVKFPGIETLLPPNAKGQNITSTLEIRECIGVEVHRASGLMA 166
                          170       180       190       200       210       220       230       240
                   ....*....|....*....|....*....|....*....|....*....|....*....|....*....|....*....|
Query_16388    166 CFLFRRCHFCKMIDADNPIGGKDGVITFENLSGDWGKGNYVIGGRTSYGSVSSAQFLRNNGGFARDGGVIGFTSYRAGES 245
Cdd:pfam09251  167 GFLFRGCHFCKMVDANNPSGGKDGIITFENLSGDWGKGNYVIGGRTSYGSVSSAQFLRNNGGFERDGGVIGFTSYRAGES 246
                          250       260       270       280       290       300       310       320
                   ....*....|....*....|....*....|....*....|....*....|....*....|....*....|....*....|
Query_16388    246 GVKTWQGSVGSSTARNYNLQFRDSVVLYPVWEGFDLGADTDMNPEDDRPGDFPFSQYPVHMLPLNHLIDNLLVRGSLGVG 325
Cdd:pfam09251  247 GVKTWQGTVGSTTSRNYNLQFRDSVVIYPVWDGFDLGADTDMNPELDRPGDYPITQYPLHQLPLNHLIDNLLVRGALGVG 326
                          330       340       350       360       370       380       390       400
                   ....*....|....*....|....*....|....*....|....*....|....*....|....*....|....*....|
Query_16388    326 FGMDGKGLYVSNITVEDCAGSGAYILAHETVFTNIAIIDTNTKNFPANQIYISGACRVNGLRLVGIRSTNEQGLTIDAPN 405
Cdd:pfam09251  327 FGMDGKGMYVSNITVEDCAGSGAYLLTHESVFTNIAIIDTNTKDFQANQIYISGACRVNGLRLIGIRSTDGQGLTIDAPN 406
                          410       420       430       440       450       460
                   ....*....|....*....|....*....|....*....|....*....|....*....|....*..
Query_16388    406 STVSGITGLVDPSRINVANLADEGLGNTRINSFNNDSAALQLRIHKLTKTLDSGAVYSHINGGPGSG 472
Cdd:pfam09251  407 STVSGITGMVDPSRINVANLAEEGLGNIRANSFGYDSAAIKLRIHKLSKTLDSGALYSHINGGAGSG 473
```

Figure S1. Alignment of amino acid sequences of the tailspike (gp65), STGO 35-1 phage, and super families and putative conserved domains of P22 tail (pfam09251);

Match sequence (*) marked in red and in blue different sequence.
