# Supplementary material for: Novel Salmonella Phage, vB_Sen_STGO-35-1, Characterization and Evaluation in Chicken Meat
Source: Microorganisms. 2022 Mar 12;10(3):606. doi: 10.3390/microorganisms10030606 (PMC8954984; doi:10.3390/microorganisms10030606)
Supplement: Supplementary file 1 [file microorganisms-10-00606-s001.zip › Figure S/Figure S2.pdf]

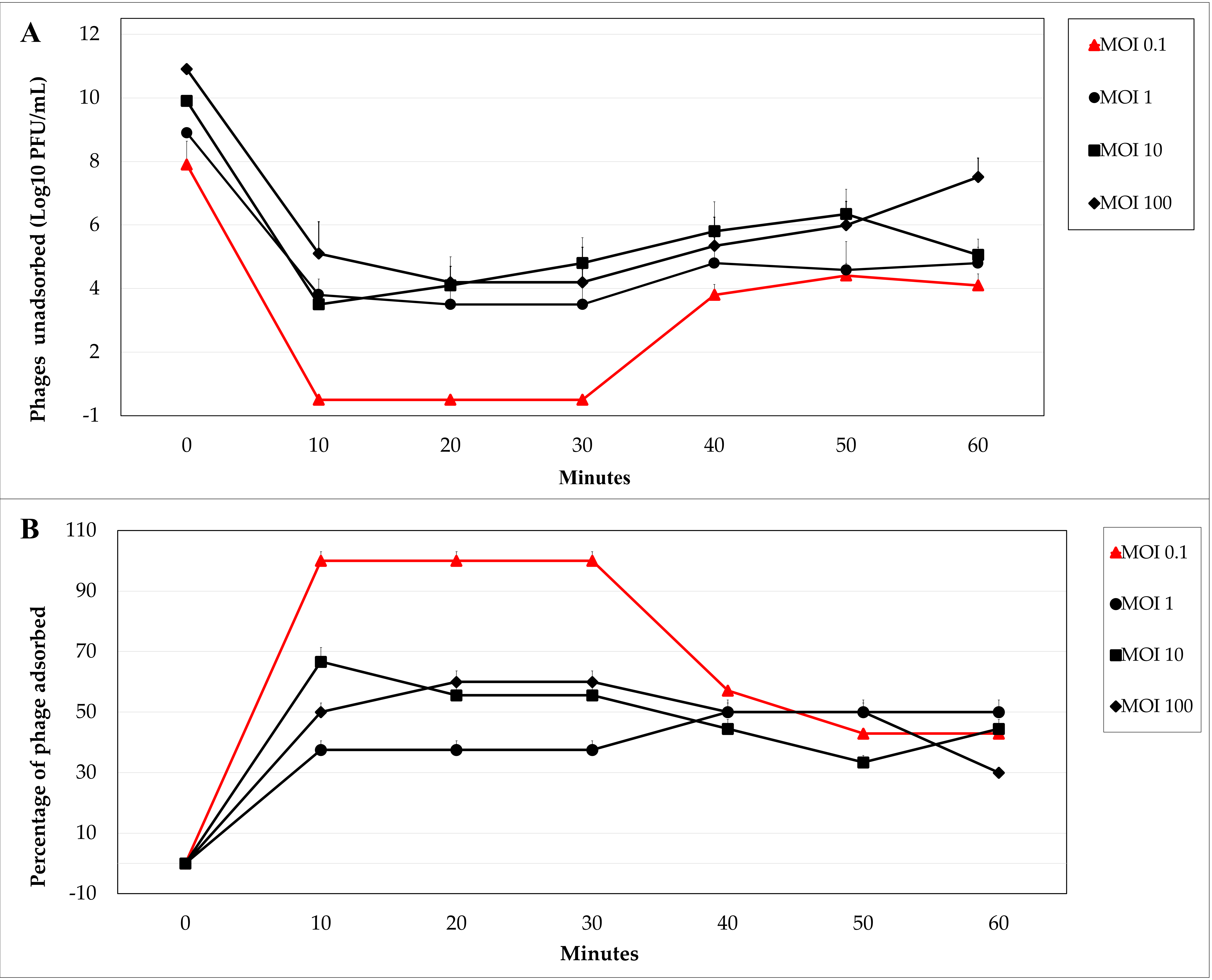

Figure S2. Adsorption rate of phage STGO-35-1 on *S.* Enteritidis DR028 strain. Assay conducted with different MOI 0.1 (▲); 1 (●) 10; (■), and 100 (◆). For this assay, samples were taken 0, 10, 20, 30, 40, 50, and 60 min after phage infection. A. The adsorption rate was calculated according to the number of PFU/mL remaining in the supernatant. B. The adsorption percentage, expressed as a percentage of the number of initial concentrations (PFU/mL) in the cultures.
