# Supplementary material for: Novel Salmonella Phage, vB_Sen_STGO-35-1, Characterization and Evaluation in Chicken Meat
Source: Microorganisms. 2022 Mar 12;10(3):606. doi: 10.3390/microorganisms10030606 (PMC8954984; doi:10.3390/microorganisms10030606)
Supplement: Supplementary file 1 [file microorganisms-10-00606-s001.zip › Figure S/Figure S4.pdf]

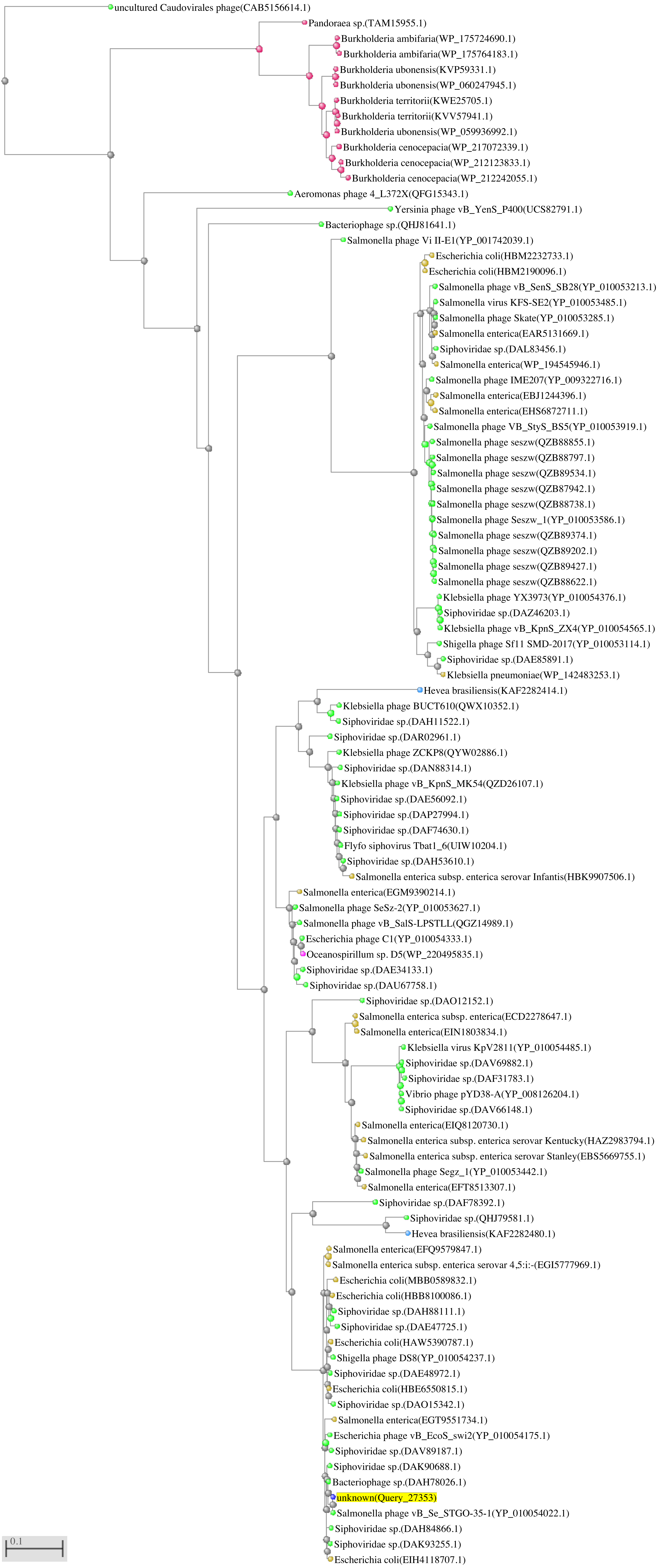
uncultured Caudovirales phage(CAB5156614.1)
Pandoraea sp.(TAM15955.1)
Burkholderia ambifaria(WP_175724690.1)
Burkholderia ambifaria(WP_175764183.1)
Burkholderia ubonensis(KVP59331.1)
Burkholderia ubonensis(WP_060247945.1)
Burkholderia territorii(KWE25705.1)
Burkholderia territorii(KVV57941.1)
Burkholderia ubonensis(WP_059936992.1)
Burkholderia cenocepacia(WP_217072339.1)
Burkholderia cenocepacia(WP_212123833.1)
Burkholderia cenocepacia(WP_212242055.1)
Aeromonas phage 4_L372X(QFG15343.1)
Yersinia phage vB_YenS_P400(UCS82791.1)
Bacteriophage sp.(QHJ81641.1)
Salmonella phage Vi II-E1(YP_001742039.1)
Escherichia coli(HBM2232733.1)
Escherichia coli(HBM2190096.1)
Salmonella phage vB_SenS_SB28(YP_010053213.1)
Salmonella virus KFS-SE2(YP_010053485.1)
Salmonella phage Skate(YP_010053285.1)
Salmonella enterica(EAR5131669.1)
Siphoviridae sp.(DAL83456.1)
Salmonella enterica(WP_194545946.1)
Salmonella phage IME207(YP_009322716.1)
Salmonella enterica(EBJ1244396.1)
Salmonella enterica(EHS6872711.1)
Salmonella phage VB_StyS_BS5(YP_010053919.1)
Salmonella phage seszw(QZB88855.1)
Salmonella phage seszw(QZB88797.1)
Salmonella phage seszw(QZB89534.1)
Salmonella phage seszw(QZB87942.1)
Salmonella phage seszw(QZB88738.1)
Salmonella phage Seszw_1(YP_010053586.1)
Salmonella phage seszw(QZB89374.1)
Salmonella phage seszw(QZB89202.1)
Salmonella phage seszw(QZB89427.1)
Salmonella phage seszw(QZB88622.1)
Klebsiella phage YX3973(YP_010054376.1)
Siphoviridae sp.(DAZ46203.1)
Klebsiella phage vB_KpnS_ZX4(YP_010054565.1)
Shigella phage Sf11 SMD-2017(YP_010053114.1)
Siphoviridae sp.(DAE85891.1)
Klebsiella pneumoniae(WP_142483253.1)
Hevea brasiliensis(KAF2282414.1)
Klebsiella phage BUCT610(QWX10352.1)
Siphoviridae sp.(DAH11522.1)
Siphoviridae sp.(DAR02961.1)
Klebsiella phage ZCKP8(QYW02886.1)
Siphoviridae sp.(DAN88314.1)
Klebsiella phage vB_KpnS_MK54(QZD26107.1)
Siphoviridae sp.(DAE56092.1)
Siphoviridae sp.(DAP27994.1)
Siphoviridae sp.(DAF74630.1)
Flyfo siphovirus Tbat1_6(UIW10204.1)
Siphoviridae sp.(DAH53610.1)
Salmonella enterica subsp. enterica serovar Infantis(HBK9907506.1)
Salmonella enterica(EGM9390214.1)
Salmonella phage SeSz-2(YP_010053627.1)
Salmonella phage vB_SalS-LPSTLL(QGZ14989.1)
Escherichia phage C1(YP_010054333.1)
Oceanospirillum sp. D5(WP_220495835.1)
Siphoviridae sp.(DAE34133.1)
Siphoviridae sp.(DAU67758.1)
Siphoviridae sp.(DAO12152.1)
Salmonella enterica subsp. enterica(ECD2278647.1)
Salmonella enterica(EIN1803834.1)
Klebsiella virus KpV2811(YP_010054485.1)
Siphoviridae sp.(DAV69882.1)
Siphoviridae sp.(DAF31783.1)
Vibrio phage pYD38-A(YP_008126204.1)
Siphoviridae sp.(DAV66148.1)
Salmonella enterica(EIQ8120730.1)
Salmonella enterica subsp. enterica serovar Kentucky(HAZ2983794.1)
Salmonella enterica subsp. enterica serovar Stanley(EBS5669755.1)
Salmonella phage Segz_1(YP_010053442.1)
Salmonella enterica(EFT8513307.1)
Siphoviridae sp.(DAF78392.1)
Siphoviridae sp.(QHJ79581.1)
Hevea brasiliensis(KAF2282480.1)
Salmonella enterica(EFQ9579847.1)
Salmonella enterica subsp. enterica serovar 4,5:i:-(EGI5777969.1)
Escherichia coli(MBB0589832.1)
Escherichia coli(HBB8100086.1)
Siphoviridae sp.(DAH88111.1)
Siphoviridae sp.(DAE47725.1)
Escherichia coli(HAW5390787.1)
Shigella phage DS8(YP_010054237.1)
Siphoviridae sp.(DAE48972.1)
Escherichia coli(HBE6550815.1)
Siphoviridae sp.(DAO15342.1)
Salmonella enterica(EGT9551734.1)
Escherichia phage vB_EcoS_swi2(YP_010054175.1)
Siphoviridae sp.(DAV89187.1)
Siphoviridae sp.(DAK90688.1)
Bacteriophage sp.(DAH78026.1)
unknown(Query_27353)
Salmonella phage vB_Se_STGO-35-1(YP_010054022.1)
Siphoviridae sp.(DAH84866.1)
Siphoviridae sp.(DAK93255.1)
Escherichia coli(EIH4118707.1)
0.1

Figure S4. The terminase phylogenetic tree was constructed to describe the closeness between amino acid sequences of long terminase from different phages. Blast names correspond to informal high-level taxonomic categories and the NCBI accession ID. Yellow squares represent of the terminal node indicating the blast name of the sequence. This analysis was performed with COBALT using the algorithm given by neighbor joining.
