# Supplementary material for: Novel Salmonella Phage, vB_Sen_STGO-35-1, Characterization and Evaluation in Chicken Meat
Source: Microorganisms. 2022 Mar 12;10(3):606. doi: 10.3390/microorganisms10030606 (PMC8954984; doi:10.3390/microorganisms10030606)
Supplement: Supplementary file 1 [file microorganisms-10-00606-s001.zip › Figure S/Figure S5.pdf]

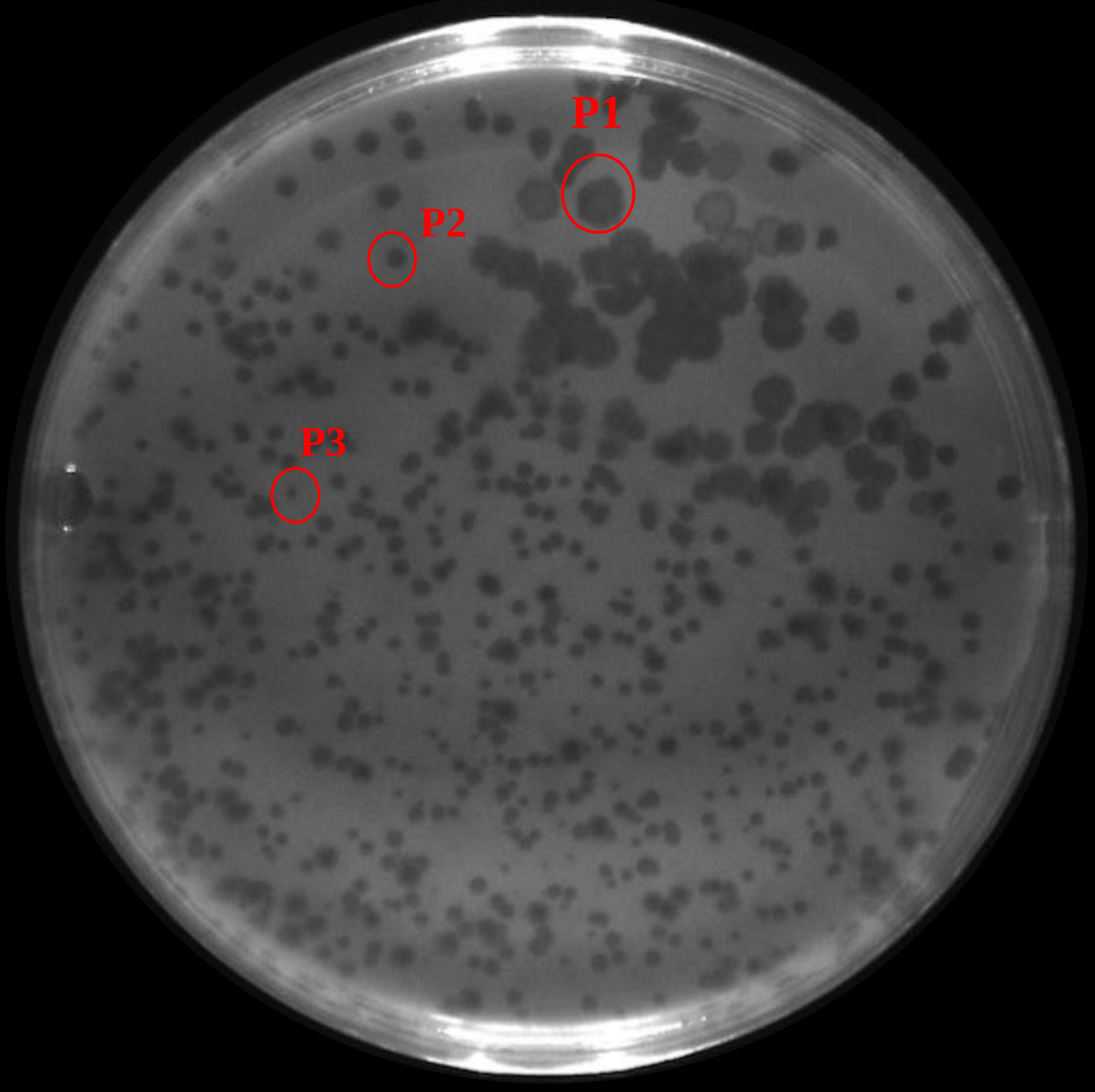

Figure S5. Morphological plaque obtained from selections of phage exposed to *S.* Enteritidis. The selected morphologically different plaques are marked in red;
