# Supplementary material for: Novel Salmonella Phage, vB_Sen_STGO-35-1, Characterization and Evaluation in Chicken Meat
Source: Microorganisms. 2022 Mar 12;10(3):606. doi: 10.3390/microorganisms10030606 (PMC8954984; doi:10.3390/microorganisms10030606)
Supplement: Supplementary file 1 [file microorganisms-10-00606-s001.zip › Table S/Table S1.pdf]

Table S1. List of *Salmonella* isolates used for phage host range characterization.

Salmonella ID[1]	Origin	Serogroup	Serovar	Phage-sensitive strains[3]
FSL A4-525	US-bovine	E1	Anatum	No
FSL R8-798	US-human	W	Weltevreden	No
FSL S5-961	US-human	C1	Virchow	No
FSL S5-369	US-human	B	Saintpaul	No
FSL R8-092	US-human	C2-C3	Corvalis	No
FSL A4-793	US-bovine	E1	Mbandaka	No
FSL S5-464	US-human	B	Stanley	No
FSL S5-373	US-human	C1	Braenderup	Yes[4]
FSL A4-737	US-bovine	B	Typhimurium	No
FSL S5-515	US-human	C2	Newport	No
FSL S5-474	US-human	C1	Montevideo	No
FSL S5-917	US-bovine	E1	Muenster	No
FSL S5-431	US-bovine	C2-C3	Kentucky	No
FSL S5-548	US-bovine	C2	Newport	No
FSL S5-370	US-human	B	Typhimurium	No
FSL S5-506	US-human	C1	Infantis	No
FSL A4-737	US-bovine	B	Typhimurium	No
FSL S5-454	US-human	D	Panama	Yes[4]
FSL R8-376	US-animal	C1	Oranienburg	No
FSL R9-1343	US-porcine	C1	Choleraesuis	No
FSL S5-667	US-human	B	Agona	Yes[4]
FSL S5-455	US-human	B	Heidelberg	No
FSL S5-406	US-human	D1	Javiana	No
FSL S5-371	US-human	D1	Enteritidis (DR028)[2]	Yes[4]
FSL S5-368	US-bovine	D1	Dublin	No
FSL S5-390	US-human	B	4,5,12:i:-	No

(1) *Salmonella* isolates were obtained from the Food Safety Laboratory (FSL; Cornell University, Ithaca, NY)
(2) Host for isolation
(3) These bacteria were sensitive to the phage vB_Sen_STGO-35-1
(4) Characterization of phage host range by spotting soft agar (0.7% TSA). Plates were incubated for 16 to 18 h at 37ºC and then examined for lysis (either present or not present) considering clean and turbid plaques= presence of lysis, and absence of plaques= no lysis. Experiments were performed in two independent replicates.
