# Supplementary material for: Novel Salmonella Phage, vB_Sen_STGO-35-1, Characterization and Evaluation in Chicken Meat
Source: Microorganisms. 2022 Mar 12;10(3):606. doi: 10.3390/microorganisms10030606 (PMC8954984; doi:10.3390/microorganisms10030606)
Supplement: Supplementary file 1 [file microorganisms-10-00606-s001.zip › Table S/Table S2.pdf]

Table S2. Transduction frequency of phage STGO 35-1.

Volume of phage Inoculation (Concentration)	Frequency of P22 HT *int* phage [(2)]	Frequency of STGO 35-1 phage [(1)]
0	0	0
1 µl (10^8 PFU/mL)	$2x10^{-6}$	0
5 µl (10^8 PFU/mL)	$6x10^{-6}$	0
20 µl (10^8 PFU/mL)	$3x10^{-5}$	0

[1] Transduction frequency was calculated as CFU/PFU.

[2] P22 HT *int* Phage donor, is a transducer phage of *Salmonella* spp. that was used as a positive control.

S. Typhimurium 14028s donor of a resistance gene.
