# Supplementary material for: Novel Salmonella Phage, vB_Sen_STGO-35-1, Characterization and Evaluation in Chicken Meat
Source: Microorganisms. 2022 Mar 12;10(3):606. doi: 10.3390/microorganisms10030606 (PMC8954984; doi:10.3390/microorganisms10030606)
Supplement: Supplementary file 1 [file microorganisms-10-00606-s001.zip › Table S/Table S3.pdf]

Table S3. Annotated CDS of wild-type *Salmonella* vB_Sen_STGO-35-1, phage, including product, predicted function, and functional category of genes.

Colour key	CDS No.	Position (nt)		Length bp	Product (b)	Predicted function	Functional Category	MS spectrometry	
		Start	Stop					Protein molecular weight (Da)	Percentage sequence coverage
	1	1	1425	1425	Phage terminase, large subunit	Endonuclease, Hydrolase, Nuclease	DNA genome packaging	–	–
	2 (c)	1458	2054	597	Hypothetical protein	Unknown	Hypothetical	–	–
	3	2228	2434	207	Hypothetical protein	Unknown	Hypothetical	–	–
	4	2438	2605	168	hypothetical protein	Unknown	Hypothetical	–	–
	5	2605	2793	189	Hypothetical protein	Unknown	Hypothetical	–	–
	6	2793	3011	219	Hypothetical protein	Unknown	Hypothetical	–	–
	7	3011	3172	162	Hypothetical protein	Unknown	Hypothetical	–	–
	8	3182	3376	195	Hypothetical protein	Unknown	Hypothetical	–	–
	9	3376	3642	267	Hypothetical protein	Unknown	Hypothetical	–	–
	10	3690	5093	1404	Capsid protein/Phage portal (connector) protein (a, b)	Genome packaging, neck/tail attachment	Structure	52.523,30	31,51%
	11	5074	6030	957	Capsid protein /Putative head assembly protein (a, b)	Assembly	Structure	35.410,90	20,80%
	12	6121	7023	903	Hypothetical protein	Unknown	Unknown	–	–
	13 (c)	7078	7377	300	Hypothetical protein	Unknown	Unknown	–	–
	14 (c)	7374	7643	270	Hypothetical protein	Unknown	Unknown	–	–
	15 (c)	7652	7795	144	Hypothetical protein	Unknown	Unknown	–	–

	16 (c)	7798	8076	279	Hypothetical protein	Unknown	Unknown	–	–
	17	8180	8458	279	Phage lysozyme (b)	Phage lysozyme	Structure	–	–
	18	8448	8912	465	Phage lysozyme R (a, b)	Unknown	Unknown	16.867,30	11,70%
	19	8909	9148	240	Putative holin	Unknown	Unknown	–	–
	20	9132	9620	489	DUF2514 family(scaffold 56 gp20) (a, b)	Unknown	Structure	17.658,20	5,56%
	21 (c)	9655	9900	246	Hypothetical protein	Unknown	Unknown	–	–
	22 (c)	10026	10334	309	Hypothetical protein	Unknown	Unknown	–	–
	23 (c)	10334	10756	423	Hypothetical protein	Unknown	Unknown	–	–
	24 (c)	10783	10977	195	Hypothetical protein/Holin (b)	Unknown	Unknown	–	–
	25	11077	11280	204	Hypothetical protein	Unknown	Unknown	–	–
	26	11291	11485	195	Hypothetical protein	Unknown	Unknown	–	–
	27	11482	11796	315	Hypothetical protein	Unknown	Unknown	–	–
	28	11793	12038	246	Restriction allevation	DNA genome metabolims	DNA genome metabolims	–	–
	29	12028	12516	489	Hypothetical protein	Unknown	Unknown	–	–
	30	12642	13022	381	Phage coat protein (cytosine-specific DNA(gp7) (b)	Unknown	Unknown	–	–
	31	13019	13330	312	Hypothetical protein	Unknown	Unknown	–	–
	32	13452	13598	147	Hypothetical protein	Unknown	Unknown	–	–
	33	13792	15078	1287	Phage coat /Capside protein (a, b)	Unknown	Structure	47.220,20	4,91%
	34	15078	15545	468	Putative capside decoration (a, b)	Unknown	Structure	15.858,90	16,10%
	35	15548	16618	1071	Phage coat protein/major capsid protein (a, b)	Unknown	Structure	39.761,20	40,72%

	36	16661	17008	348	Hypothetical protein	Unknown	Unknown	–	–
	37	17001	17198	198	Hypothetical protein	Unknown	Unknown	–	–
	38	17239	18057	819	Hypothetical protein (a, b)	Unknown	Structure	28.848,30	24,60%
	39 (c)	18085	18444	360	Phage protein	Unknown	Unknown	–	–
	40 (c)	18444	18983	540	Hypothetical protein	Unknown	DNA genome metabolims	–	–
	41 (c)	18983	19156	174	DNA polymerase III/ putative structural protein (b)	Unknown	Unknown	–	–
	42 (c)	19153	19398	246	Hypothetical protein	Unknown	Unknown	–	–
	43 (c)	19395	20207	813	Phage protein/ putative trascriptional regulator (b)	DNA genome metabolims	DNA genome metabolims	–	–
	44 (c)	20204	20485	282	Hypothetical protein	Unknown	Unknown	–	–
	45 (c)	20487	20678	192	Hypothetical protein/ phage fibritin(wac) protein	Unknown	Unknown	–	–
	46 (c)	20682	21305	624	Phage protein/ Phage EaC protein (b)	Unknown	Unknown	–	–
	47	21423	21542	120	Hypothetical protein	Unknown	Unknown	–	–
	48	21542	21982	441	Hypothetical protein	Unknown	Unknown	–	–
	49	22015	22299	285	Hypothetical protein/ phage tail tape measure (b)	Unknown	Unknown	–	–
	50	22316	22672	357	Phage protein	Unknown	Unknown	–	–
	51	22669	23010	342	Minor capsid protein (gp51) (a, b)	Packaging of viral DNA	Structure	12.385,00	9,73%
	52	23010	23408	399	Minor capsid protein (gp52) (a, b)	Packaging of viral DNA	Structure	14.814,80	15,20%
	53	23405	23779	375	Minor capsid protein (gp53) (a, b)	Unknown	Structure	14.009,10	9,68%
	54 (c)	23811	23884	74	tRNA-Met-CAT	Unknown	tRNA	-	-
	55	24475	25191	717	Phage fibritin (wac)/Phage tail tube (a, b)	Chaperone involved in tail fiber contractile mechanical	Structure	24.988,40	20,17%

	56 (c)	25427	25729	303	Hypothetical protein /Phage NinX	Unknown	Unknown	–	–
	57 (c)	25726	25869	144	Hypothetical protein	Unknown	Unknown	–	–
	58 (c)	25866	26060	195	Hypothetical protein	Unknown	Unknown	–	–
	59 (c)	26093	26386	294	Hypothetical protein	Unknown	Unknown	–	–
	60	26500	27213	714	Hypothetical protein / Tape measure-2 (a, b)	Unknown	Unknown	–	–
	61	27206	29548	2343	Phage tail tape measure/ Phage minor tail (a, b)	Stopping the tail tube polymerization	Structure	81.265,10	31,30%
	62	29548	30021	474	Tail tip L/ Phage minor tail (a, b)	Unknown	Structure	18.095,10	36.30%
	63	30021	30491	471	Putative structural protein/tail tip (b)	Unknown	Structure	17.705,90	16,00%
	64	30484	30933	450	Putative phage tail /tail tip assembly k (a, b)	Unknown	Unknown	–	–
	65	30887	33349	2463	Putative phage tail/ P22 Tailspike protein (a, b)	Neck/tail attachment	Structure	91.351,30	10,40%
	66	33389	35416	2028	Tail-spike/ tail tip assembly k (b)	Tail protein	Structure	72.772,30	35,82%
	67 (c)	35712	36194	483	Single-stranded DNA binding protein	DNA binding protein	DNA genome metabolism	–	–
	68 (c)	36198	37238	1041	Hipothetical protein/Phage associated recombinase (b)	Recombinase	DNA genome metabolism	–	–
	69 (c)	37256	38194	939	Hypothetical protein/ Exodeoxyribonuclease VIII (b)	Unknown	DNA genome metabolism	–	–
	70 (c)	38191	38463	273	Hypothetical protein/tail fibers protein (b)	Unknown	Unknown	–	–
	71 (c)	38501	38926	426	Hypothetical protein	Unknown	Unknown	–	–
	72 (c)	38928	40853	1926	DNA helicase/Putative ATP dependent helicase (b)	DNA and RNA metabolism	Structure	–	–
	73	40929	41882	954	DNA primase/helicase (b)	DNA and RNA metabolism	Structure	–	–
	74 (c)	41911	42216	306	Hypothetical protein	Unknown	Unknown	–	–

	75 (c)	42226	42417	192	Hypothetical protein	Unknown	Unknown	–	–
	76 (c)	42414	43967	1554	DUF3987 protein of unknown function	Unknown	Unknown	–	–
	77 (c)	43967	44185	219	Hypothetical protein	Unknown	Unknown	–	–
	78	44641	44730	90	Hypothetical protein	Unknown	Unknown	–	–
	79	44816	45010	195	Hypothetical protein	Unknown	Unknown	–	–
	80	45065	45187	123	Hypothetical protein	Unknown	Unknown	–	–
	81	45191	45373	183	Hypothetical protein	Unknown	Unknown	–	–
	82	45410	45643	234	Hypothetical protein	Unknown	Unknown	–	–
	83	45640	46032	393	Hypothetical protein	Unknown	Unknown	–	–
	84	46096	46347	252	Hypothetical protein	Unknown	Unknown	–	–
	85	46340	46456	117	Hypothetical protein	Unknown	Unknown	–	–
	86	46449	46628	180	Hypothetical protein/holin (b)	Unknown	Unknown	–	–
	87	46615	46788	174	Hypothetical protein/DUF5681 (b)	Unknown	Unknown	–	–
	88	46936	47355	420	DUF5681(a, b)	Unknown	Structure	15.622,80	19,40%
	89	47348	47483	135	Hypothetical protein	Unknown	Unknown	–	–

(a) Identification by mass spectrometry, (b) HHpred prediction, (c) Reverse gene drive. The color keys (previously used in the genetic map, Figure 3) correspond to: Terminase (yellow) capsid proteins (green); lysozyme (black); CDSs and hypothetical proteins (grey); tail proteins (red).
