# Supplementary material for: Novel Salmonella Phage, vB_Sen_STGO-35-1, Characterization and Evaluation in Chicken Meat
Source: Microorganisms. 2022 Mar 12;10(3):606. doi: 10.3390/microorganisms10030606 (PMC8954984; doi:10.3390/microorganisms10030606)
Supplement: Supplementary file 1 [file microorganisms-10-00606-s001.zip › Table S/Table S4.pdf]

Table S4. Relatedness and taxonomical classification of similar *Siphoviridae* sequence of phages obtained by JSpeciesWS.

Genome	Accession	ANIb [%]	Aligned [%]	Aligned [bp]	ANIb x Aligned [%]
Klebsiella phage vB_KpnS_ZX4	NC_054654.1	72.50	46.82	1402	35.34
Klebsiella phage BUCT541	MZ836210.1	72.91	42.79	2338	31.19
Shigella phage Sf11_SMD-2017	NC_054636.1	83.45	43.29	20556	36.13
Salmonella_virus_VSt472	NC_054644.1	83.96	53.57	25436	44.98
Salmonella phage Skate	NC_054639.1	84.33	55.41	26310	46.73
Salmonella phage IME207	KX523699.2	84.40	49.37	23442	41.67
**Salmonella phage KFS-SE2(*)**	NC_054641.1	**84.81**	**53.22**	**25272**	**45.14**
Salmonella phage vB_SenS_SB28	NC_054638.1	84.96	54.28	14507	33.62
Salmonella phage VB_StyS_BS5	NC_054646.1	85.47	63.02	17309	53.86
Salmonella phage_LPST10	NC_054645.1	85.72	57.02	27074	48.88
Salmonella phage Vi II-E1	AM491472.1	85.78	45.43	21570	38.97
**Salmonella phage_64795_sal3(*)**	KX017520.1	**85.92**	**49.43**	**23471**	**42.47**
Salmonella phage vB_SalS-LPSTLL	MN737551.1	86.76	62.07	17702	53.85
Salmonella phage seszw	MZ375232.1	86.79	61.20	18800	53.11
E. coli phage vB_EcoS-Sa179lw	NC_054637.1	86.97	39.58	18795	34.42
Escherichia phage vB_EcoS_swi2	NC_054649.1	87.23	68.17	22434	59.46
Salmonella phage D10	MZ489634.1	87.34	69.28	21530	60.50
Escherichia phage C1	NC_054651.1	87.84	56.95	27042	50.02
Shigella phage DS8	NC_054650.1	87.89	52.39	24877	46.05
**Salmonella phage Akira(*)**	NC_054647.1	**88.53**	**54.15**	**25712**	**47.94**

In gray, the phages closest to the study phage STGO-35-1 are highlighted.
(*) These phages are represented within the phylogenetic analysis in Figure 4 in the large cluster STGO-35-1.
