# Supplementary figures and images for: Novel Salmonella Phage, vB_Sen_STGO-35-1, Characterization and Evaluation in Chicken Meat
Source: Microorganisms. 2022 Mar 12;10(3):606. doi: 10.3390/microorganisms10030606 (PMC8954984; doi:10.3390/microorganisms10030606)

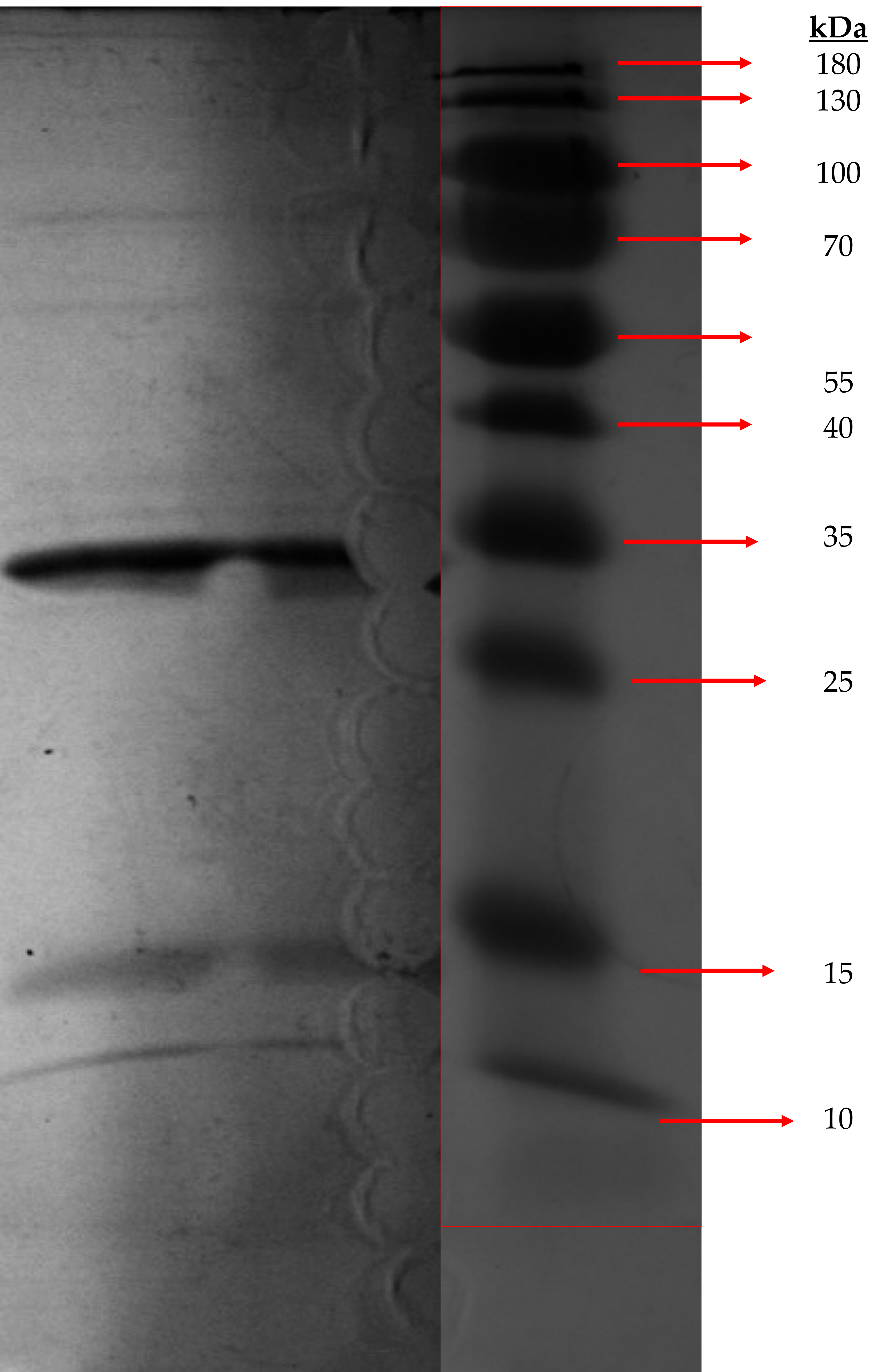

**Figure S3.** SDS-PAGE analysis of phage structural proteins.

Supplement: Supplementary file 1 [file microorganisms-10-00606-s001.zip › Figure S/Figure S3.pdf]
